# Supplementary material for: Digitally delivered, systemically challenged: A qualitative study of health system readiness for digital care
Source: PLOS Digit Health. 2026 Jan 6;5(1):e0001193. doi: 10.1371/journal.pdig.0001193 (PMC12773818; doi:10.1371/journal.pdig.0001193)
Supplement: S1 Appendix — (DOCX) [file pdig.0001193.s001.docx]

**S1 Appendix: Interview Guide**

***Evaluation of attitudes towards Test-to-Treat and decentralized Care Initiatives***

**Introduction/Verbal Consent:**

Hello, my name is ________, and I am a part of the research team at [lab conducting the study].

We are speaking with healthcare leaders about their perspectives on home-based care that patients receive from third-party and external programs. These may include government-sponsored home-based testing and treatment initiatives (e.g. Home Test to Treat) or for-profit (e.g., Amazon One Medical) efforts that reach directly to the patient.

We recently led the research evaluation of the largest nationwide home-based COVID-19, and flu testing and treatment program sponsored by the federal government through the NIH and Administration for Strategic Preparedness and Response (ASPR) branches of Health and Human Services. We observed that this program enrolled more than 80,000 individuals, distributed home antigen and PCR tests for COVID-19 and/or Influenza A/B to more than 40,000 individuals, and more than 8,000 people who tested positive elected to receive care from the program, resulting in 7,000 individuals being prescribed medications. However, none of this care information is available to the patient’s routine care providers unless the patients themselves inform their primary care providers. We believe this fragmentation of care can have consequences for the existing healthcare ecosystem and want to better understand its impact.

We are interviewing leaders from a healthcare organization who are responsible for making or informing system-level decisions. Interviewee roles include but are not limited to Chief Executive Officers, Chief Finance Officers, Chief Medical Officers, Chief Innovation Officers, Chief Quality Officers, and Medical Directors of ambulatory clinics.

Your participation in this interview is completely voluntary, and you can choose not to participate or answer any questions that I may ask you. The information we discuss will be confidential. Are you still interested in participating? The interview should take no more than 45 - 60 minutes. Okay, let’s begin.

**Welcome Reminders:**

- Thank you again for coming and taking the time to participate in this project
- This is a qualitative study where the structured interviews will be analyzed for common themes and ideas between different participants. There are no right or wrong answers.
- We are recording the conversation so that we can transcribe it later
- There is no silly or wrong question or response
- When we publish this project, no identifying information will be published regarding any individual person.
- You may choose not to answer any question at any time, and you may leave at any time.
- Your participation is entirely voluntary
- You may decline to answer any of the questions if you like

**What we are studying (Major Domains of Focus)**

- What are ways that health systems/payors can be involved in national home tests to treat or other home-based healthcare delivery programs to avoid fragmentation of care?
- What is your vision of the role of home-based care in your health system?
  - If appropriate: how are you addressing the expansion of decentralized care?
- How do you select and implement digital health interventions?

1. **Health system integration for future programs**

- *Interviewer: Evoke the NIH/ASPR Home Test to Treat program and how we enrolled 100K people, gave telehealth to 10K, and treatment to 8K people.*
  - - We noted that we are siloing care for these patients. Patients themselves are interested but our goal is to not fragment care.
- Do you see a role that your health system can/should play in the future deployment of home-based testing and treatment programs?
  - If yes: what opportunities do home-based care models offer for the health system
- How would such programs improve the health/healthcare delivery of patients in your health system?
  - Probe: How do you envision this unfolding in such a way that care is not fragmented?
- Question to the system: [slide to help with the discussion]
  - What circumstances would prompt your health system to integrate with a home-based testing and treatment program?
    - What will it take for you to be involved from a resource and cost perspective?
    - How complicated would adoption be for your system?
      - Probe: Please consider the length of time to adoption, intricacy, and number of steps involved, and whether the intervention reflects a clear departure from previous practices.
      - Probe: What costs will be incurred to implement a home-based testing and treatment program?
      - Probe: What costs will be considered when deciding to implement the intervention?
    - Have you implemented something like the Home Test to Treat program? If yes, how did they decide to implement it? If not, what do you think you haven’t yet?
      - Probe: is this something you think is valuable?
    - How activated are you currently to integrate a home-based testing and treatment program in your health system if given the opportunity?
    - What would move the needle on your activation?
      - PROBE: If you were asked to integrate a federal home test to treat program within your system so that either

1. (least involved) you can refer your patients to the program with no future involvement,
2. (b-1) (moderately involved) refer patients to the program, share EMR data with the program, and
3. **(b-2) Receive encounter details from the program back to your EMR or**
4. Be an in-person option for the home test-to-treat program.
   1. In-person clinical assessment
   2. Help with medication delivery

- How will the infrastructure of your organization (social architecture, age, maturity, size, or physical layout) affect the implementation of home-based care
- How do you think your organization's culture (general beliefs, values, assumptions that people embrace) will affect the implementation of the intervention?

1. **Decentralized care roadmap**

- What are other areas where you think this idea of home-based care can help solve problems that you are facing?
  - Probe: Specific care gaps (primary care, acute care, etc.)
  - Probe: Certain populations- homebound elderly etc.
- [Interviewer, give some case examples of existing decentralized care programs: Hepatitis C test to treat, Telepulmonary rehabilitation, Amazon/industry-led programs, MIH]
- Probe: How well do you think home-based care will meet the needs of the individuals served by your organization?
  - Probe to Probe: In what ways will the intervention meet their needs?
    - E.g. improved access to services? Reduced wait times? Help with self-management? Reduced travel time and expense?
  - What barriers will the individuals served by your organization face to participating in the intervention?
  - What advantages does home-based care have compared to existing programs in your system?
  - How do home-based models compare to other alternatives that may have been considered or that you know about?
  - What are some efforts for thinking about new ways to deliver care? OR what does decentralized (home-based) care mean to you?
  - What are some of the major problems that they think can be solved with decentralized (home-based) care
- What are among your biggest barriers to implementing decentralized / home-based care
  - Which other system-level stakeholders would need to be on board from your organization?
  - Are there specific adaptations that would need to be made to decentralized models to make them fit well in your system ( the interviewer can probe: cultural or language concerns, and technology concerns)
  - What kind of local, state, or national performance measures, policies, regulations, or guidelines influenced the decision to implement the intervention?
  - Are there some types of models that are easier to implement than others

1. **Digital Health Interventions**

- Overall, what is your vision of the role of home-based care in your health system?
- How do you select digital health interventions and what is your process for implementing them
