# Supplementary material for: Digitally delivered, systemically challenged: A qualitative study of health system readiness for digital care
Source: PLOS Digit Health. 2026 Jan 6;5(1):e0001193. doi: 10.1371/journal.pdig.0001193 (PMC12773818; doi:10.1371/journal.pdig.0001193)
Supplement: Attachment — Submitted filename: PLOS Digital Health-reviewers comment(2).docx [file pdig.0001193.s002.docx]

**Manuscript ID:** PDIG-D-25-00455
**Title:** Digitally Delivered, Systemically Challenged: A Qualitative Study of Health System Readiness for Digital Care

**Overall Evaluation**

This qualitative study provides valuable insights into the facilitators and barriers of implementing digital health technologies (DHTs) within diverse U.S. health systems. The manuscript is methodologically sound and clearly structured, employing the CFIR and CENTER-IT frameworks to guide data collection and analysis. The inclusion of senior system-level stakeholders enhances the policy relevance and practical significance of the findings.

However, several issues regarding conceptual clarity, methodological transparency, and stylistic consistency should be addressed before publication. Once revised, the paper will make a meaningful contribution to the literature on digital health implementation readiness.

**Major Comments**

1. **Clarify the conceptual relationship between CENTER-IT and CFIR (Methods, Lines 96–109).**
The description of how CENTER-IT relates to CFIR is somewhat dense. Readers unfamiliar with CENTER-IT may not understand its unique contribution.
Recommendation: Add a concise explanation or visual summary differentiating CENTER-IT as a participatory adaptation methodology grounded in CFIR, emphasizing its multi-level stakeholder engagement.

**2. Improve sampling transparency and reflexivity (Methods, Lines 117–123, 160–166).**
Details on how participants were identified and diversity ensured are limited. Reflexivity regarding the physician interviewers should also be acknowledged.
Recommendation: Clarify recruitment rationale and diversity (academic vs. community systems) and include a brief reflexivity statement describing how investigators’ professional backgrounds may have influenced data interpretation.

**3. Deepen analytical interpretation in Results (Themes 1–2)**
Quotations are rich but occasionally overshadow analysis. The link between data and CFIR constructs could be more explicit.
Recommendation: Strengthen interpretive commentary connecting participant quotes to CFIR constructs (e.g., *complexity*, *compatibility*) and ERIC strategies to highlight practical implications.

**4. Expand discussion on policy and economic implications (Discussion, Lines 347–372).**
Reimbursement and regulatory barriers are described but not fully analyzed.
Recommendation: Link financial disincentives to implementation outcomes (e.g., scalability, sustainability) and situate findings within health-economic and policy implementation literature (e.g., Cresswell 2013; Greenhalgh 2017).

**5. Ensure terminology and stylistic consistency**
Terms such as *digital health tools*, *digital solutions*, and *digital health technologies* are used interchangeably.
Recommendation: Adopt a single term—preferably *Digital Health Technologies (DHTs)*—throughout the manuscript.

**Minor Comments**

1. Limitations: Please note that frontline clinicians and patients were not included in the study sample.
2. Methods – Setting:

Original: “The study staff was based at an urban academic tertiary care medical center in Massachusetts that serves urban, suburban, and rural communities.”

Issue: The verb “was” should be changed to “were” because the subject staff is plural.

3. Methods – Participant Recruitment:

Original: “expanded this list during recruitment..8, 17, 20”

Issue: There is a typographical error (double period “..8”), and the placement of the reference numbers is incorrect. Please correct the punctuation and reference positioning.

4. References:
   - Remove the residual citation marker {Moser, 2018 #74}.
   - Correct capitalization: “krippendorff K.” → “Krippendorff K.”
   - Correct the journal title: “he Qualitative Report” → “The Qualitative Report.”

**Overall Recommendation**
**Recommendation: Major Revision**
This manuscript addresses a timely and important topic with strong methodological potential. With clearer theoretical positioning, enhanced reflexivity, and consistent terminology, the paper will make a valuable contribution to understanding system-level readiness for digital health implementation.
