# Supplementary material for: Digitally delivered, systemically challenged: A qualitative study of health system readiness for digital care
Source: PLOS Digit Health. 2026 Jan 6;5(1):e0001193. doi: 10.1371/journal.pdig.0001193 (PMC12773818; doi:10.1371/journal.pdig.0001193)
Supplement: Attachment — Submitted filename: Response to Reviewers PLOS Dig Health.docx [file pdig.0001193.s004.docx]

**November 24, 2025**

**Dear Dr. Ghasemi,**

**We sincerely appreciate your time and expertise, as well as those of our reviewers. We are grateful for their thoughtful, critical reviews and believe that responding to their suggestions has significantly strengthened the manuscript. Below, please find a line-by-line response to the reviewers’ comments and the revisions made in response to their suggestions. For clarity, the authors’ responses and actions taken have been bolded. We hope that you find the manuscript substantially improved and suitable for publication.**

1. Does this manuscript meet PLOS Digital Health’s [publication criteria](https://track.editorialmanager.com/CL0/https:%2F%2Fjournals.plos.org%2Fdigitalhealth%2Fs%2Fjournal-information%23loc-criteria-for-publication/1/010f019ab4a7756c-5e835028-2243-445c-9e3d-681d88539be5-000000/sxUWRTXTKz3_ONzhTYnf_DdewDn7TXgehVODXEKkIxY=238)? Is the manuscript technically sound, and do the data support the conclusions? The manuscript must describe methodologically and ethically rigorous research with conclusions that are appropriately drawn based on the data presented.

Reviewer #1: Yes

Reviewer #2: Yes

**Author Response: The authors are gratified that the reviewers found the manuscript technically and methodologically sound**

**Action Taken: No action taken**

2. Has the statistical analysis been performed appropriately and rigorously?

Reviewer #1: Yes

Reviewer #2: Yes

**Author Response: The authors are gratified that the reviewers found the analysis rigorous**

**Action Taken: No action taken**

3. Have the authors made all data underlying the findings in their manuscript fully available (please refer to the Data Availability Statement at the start of the manuscript PDF file)?

Reviewer #1: Yes

Reviewer #2: Yes

**Author Response: The authors agree with the reviewers that all data associated with the manuscript have been made accessible to readers**

**Action Taken: No action taken**

4. Is the manuscript presented in an intelligible fashion and written in standard English?

PLOS Digital Health does not copyedit accepted manuscripts, so the language in submitted articles must be clear, correct, and unambiguous. Any typographical or grammatical errors should be corrected at revision, so please note any specific errors here.

Reviewer #1: Yes

Reviewer #2: Yes

**Author Response: The authors are gratified that the reviewers found the writing clear, correct, and readable.**

**Action Taken: See below for individual responses to any identified typographical errors.**

Reviewer #1: Overall Evaluation

This qualitative study provides valuable insights into the facilitators and barriers of implementing digital health technologies (DHTs) within diverse U.S. health systems. The manuscript is methodologically sound and clearly structured, employing the CFIR and CENTER-IT frameworks to guide data collection and analysis. The inclusion of senior system-level stakeholders enhances the policy relevance and practical significance of the findings.
However, several issues regarding conceptual clarity, methodological transparency, and stylistic consistency should be addressed before publication. Once revised, the paper will make a meaningful contribution to the literature on digital health implementation readiness.

**Author Response: The authors are gratified that the reviewer found the project valuable and meaningful. We have carefully revised the manuscript to improve issues related to conceptual clarity, methodological transparency, and stylistic consistency as suggested by the reviewer.**

**Action Taken: Please see below for actions taken in response to individual comments**

Major Comments

1. Clarify the conceptual relationship between CENTER-IT and CFIR (Methods, Lines 96–109).
The description of how CENTER-IT relates to CFIR is somewhat dense. Readers unfamiliar with CENTER-IT may not understand its unique contribution.

Recommendation: Add a concise explanation or visual summary differentiating CENTER-IT as a participatory adaptation methodology grounded in CFIR, emphasizing its multi-level stakeholder engagement.

**Author Response: The authors agree with the reviewer that readers may benefit from a clearer explanation of how CENTER-IT integrated CFIR, particularly if they are not familiar with these tools.**

**Action Taken: We have revised the Methods section to briefly describe CENTER-IT as an interactive methodology that uses CFIR as its determinant framework to help readers understand how the framework (CFIR) and the methodological strategy (CENTER-IT) are related. We have also simplified the description to improve accessibility for readers who may be less familiar with CENTER-IT and/or implementation frameworks like CFIR.**

2. Improve sampling transparency and reflexivity (Methods, Lines 117–123, 160–166).
Details on how participants were identified and diversity ensured are limited. Reflexivity regarding the physician interviewers should also be acknowledged.
Recommendation: Clarify recruitment rationale and diversity (academic vs. community systems) and include a brief reflexivity statement describing how investigators’ professional backgrounds may have influenced data interpretation.

**Author Response: The authors agree with the reviewer’s suggestion that additional clarity regarding the sampling strategy and a more explicit reflexivity statement would strengthen methodological transparency.**

**Action Taken: We have expanded the Methods to clarify how participants were identified, our rationale for selecting stakeholders across academic and community settings, and steps taken to ensure diversity in role, perspective, and system contexts. We also added a brief reflexivity statement acknowledging that members of the research team, including physician interviewers with experience in digital care delivery and implementation, may have influenced data interpretation and how this was accounted for during analysis.**

3. Deepen analytical interpretation in Results (Themes 1–2)
Quotations are rich but occasionally overshadow analysis. The link between data and CFIR constructs could be more explicit.
Recommendation: Strengthen interpretive commentary connecting participant quotes to CFIR constructs (e.g., complexity, compatibility) and ERIC strategies to highlight practical implications.

**Author Response: The authors agree with the reviewer that the Results section would benefit from deeper commentary linking participant perspectives to specific CFIR constructs and relevant ERIC implementation strategies.**

**Action Taken: We have revised Themes 1 and 2 to more clearly articulate how the qualitative data reflect constructs, including complexity, compatibility, workflow integration, and external policy and incentives. We also added brief statements highlighting which ERIC strategies naturally align with the barriers and facilitators described by participants.**

4. Expand discussion on policy and economic implications (Discussion, Lines 347–372).
Reimbursement and regulatory barriers are described but not fully analyzed.
Recommendation: Link financial disincentives to implementation outcomes (e.g., scalability, sustainability) and situate findings within health-economic and policy implementation literature (e.g., Cresswell 2013; Greenhalgh 2017).

**Author Response: The authors agree with the reviewer that barriers to reimbursement, as well as identified regulatory obstacles, warrant rigorous analysis to clarify their implications for implementation of DHTs and key outcomes, including scalability/sustainability. They understand the reviewer’s concern that these themes are very important but were not fully analyzed in the discussion.**

**Action Taken: We have expanded the discussion to more explicitly connect financial barriers to core implementation constructs, including organizational readiness, long-term maintenance, and system-level adoption of DHTs. We also incorporated perspectives from the health-economic and digital transformation manuscripts we previously cited to better contextualize how policy and payment structure misalignment shapes the implementation trajectories of DHTs**.

5. Ensure terminology and stylistic consistency
Terms such as digital health tools, digital solutions, and digital health technologies are used interchangeably. Recommendation: Adopt a single term—preferably Digital Health Technologies (DHTs)—throughout the manuscript.

**Author Response: The authors agree with the reviewer that the term “digital health technology” needs to be used consistently throughout the manuscript.**

**Action Taken: The term “Digital Health Technology” has been standardized throughout the manuscript and abbreviated where appropriate. Similar terms such as “digital health solutions” have been removed.**

Minor Comments
1. Limitations: Please note that frontline clinicians and patients were not included in the study sample.

**Author Response: The authors agree with the reviewer that perspectives from frontline clinicians and patients are not included as part of this focused project, and that this decision should be noted as a limitation of its scope.**

**Action Taken: The limitations were revised to note that frontline clinicians, patients, and representatives from international settings were not included as participants.**

2. Methods – Setting:
Original: “The study staff was based at an urban academic tertiary care medical center in Massachusetts that serves urban, suburban, and rural communities.”
Issue: The verb “was” should be changed to “were” because the subject staff is plural.

**Author Response: The authors regret this typographical error and thank the reviewer for catching it.**

**Action Taken: The referenced sentence has been revised as suggested**

3. Methods – Participant Recruitment:
Original: “expanded this list during recruitment..8, 17, 20”
Issue: There is a typographical error (double period “..8”), and the placement of the reference numbers is incorrect. Please correct the punctuation and reference positioning.

**Author Response: The authors regret this typographical error and thank the reviewer for catching it.**

**Action Taken: The referenced sentence has been revised as suggested**

4. References:
o Remove the residual citation marker {Moser, 2018 #74}.

**Author Response: The authors regret this typographical error and thank the reviewer for catching it.**

**Action Taken: The reference has been revised as suggested**

Correct capitalization: “krippendorff K.” → “Krippendorff K.”

**Author Response: The authors regret this typographical error and thank the reviewer for catching it.**

**Action Taken: The reference has been revised as suggested**

Correct the journal title: “he Qualitative Report” → “The Qualitative Report.”

**Author Response: The authors regret this typographical error and thank the reviewer for catching it.**

**Action Taken: The reference has been revised as suggested**

Overall Recommendation: Major Revision
This manuscript addresses a timely and important topic with strong methodological potential. With clearer theoretical positioning, enhanced reflexivity, and consistent terminology, the paper will make a valuable contribution to understanding system-level readiness for digital health implementation.

**Author Response: We thank the reviewer for the thoughtful, critical review. We have made numerous revisions to address her/his suggestions and believe that these changes have improved the manuscript**

**Action Taken: Please see individual responses above**

Reviewer #2: This manuscript presents a timely and well-conducted qualitative study exploring health system leaders’ perspectives on the implementation of digital health technologies (DHTs) across diverse U.S. settings. The research addresses a critical gap in implementation science by focusing on systemic readiness—rather than just technological feasibility—and offers nuanced insights into the interplay between clinical workflows, equity considerations, policy constraints, and financial sustainability. The use of the Consolidated Framework for Implementation Research (CFIR) and the CENTER-IT methodology strengthens the rigor and relevance of the findings. The paper is clearly written, thematically rich, and contributes meaningfully to ongoing discourse about scaling digital health equitably and effectively.

**Author Response: The authors are gratified that the reviewer found the project valuable and meaningful.**

**Action Taken: Please see below for actions taken in response to individual comments**

Suggestions for Revision

1. Clarify the Role of the “Home Test to Treat” Example

The manuscript introduces the Home Test to Treat program as a concrete anchor for interviews. However, its role in shaping responses could be more explicitly discussed: Consider adding a brief methodological note explaining whether participants’ views were primarily shaped by this example or generalized beyond it. In the Discussion, briefly reflect on whether findings might differ if a different DHT (e.g., remote monitoring for chronic disease) had been used as the prompt.

**Author Response: The authors agree with the reviewer that the role of the Home Test to Treat model as an anchoring example for the qualitative interviews could be described more explicitly as to how it impacted participant responses.**

**Action Taken: We added clarification in the Methods section stating that Home Test to Treat was used as a concrete reference point to facilitate discussion, but participants regularly broadened their reflections to digital health more generally. We also expanded the Discussion (within the limitations) to acknowledge that using a different digital health technology, such as chronic disease remote monitoring, as an anchoring example might have elicited different participant responses.**

2. Expand on Equity Considerations
While disparities related to broadband access and digital literacy are noted, the analysis could deepen its engagement with structural inequities:

**Author Response: The authors thank the reviewer for this important observation. We agree with the reviewer that our initial discussion of digital disparities focused primarily on broadband access and digital literacy in the results and discussion but did not fully articulate how these challenges are rooted in broader structural inequities or how they relate to broader system readiness for DHTs.**

**Action Taken: We have expanded both the Results and Discussion sections to explicitly connect participants’ concerns to upstream structural determinants, including historical underinvestment in communities already impacted by determinants of health that influence healthcare access, structural racism, neighborhood socioeconomic disadvantage, and institutional policies shaping digital infrastructure. We also elaborated on how these structural factors shape organizational readiness, perceived feasibility of digital care models, and the potential for digital implementation to exacerbate inequities without intentional mitigation strategies.**

Explicitly connect “techquity” concerns to social determinants of health (e.g., income, education, race/ethnicity).
Consider citing recent frameworks like the Digital Health Equity Framework (Richardson et al., npj Digit Med 2022) to strengthen conceptual grounding.

**Author Response: The authors agree with the reviewer that concerns related to “techquity” should be more explicitly grounded in the broader context of social determinants of health that impact access to, and the use of, DHTs.**

**Action Taken: We have revised the manuscript to clarify that inequities in income, education, digital literacy, and structural racism may shape patients’ ability to access and benefit from digital health tools. We have, as suggested, also more explicitly incorporated the Digital Health Equity Framework (Richardson et al., npj Digital Medicine, 2022) and used this framework to situate our discussion of DHTs within the determinants that influence digital access, adoption, and outcomes.**

3. Refine Terminology Consistency
The term “digital health technologies (DHTs)” is well-defined, but at times the manuscript uses “digital tools,” “digital solutions,” and “decentralized care” interchangeably. Ensure consistent use of core terminology, especially in the Results and Discussion.
Clarify early on whether “integration” refers specifically to EHR interoperability or broader workflow/system alignment.

**Author Response: The authors agree with the reviewer that the term “digital health technology” needs to be used consistently throughout the manuscript. We also agree that a clarification of what the term “integration” means concerning DHTs would help contextualize the manuscript.**

**Action Taken: The term “Digital Health Technology” has been standardized throughout the manuscript and abbreviated where appropriate. Similar terms such as “digital health solutions” have been removed. We have also revised the methods to include a clear definition of integration that notes: “In this context, “integration” of DHTs refers not only to technical EHR interoperability but also to the broader alignment of workflows, teams, and system processes needed for seamless clinical use.”**

4. Address Limitations More Fully
The current limitations section appropriately notes the focus on senior leaders. Consider adding:

Potential bias from non-response (1 of 13 approached declined); while minimal, it’s worth acknowledging.
The absence of patient or frontline clinician voices limits insight into end-user experience—a point that could be tied to future research directions.

**Author Response: The authors agree with the reviewer that the lack of end-user participants in this study, as well as the potential for sampling bias, are important limitations that should be explicitly acknowledged.**

**Action Taken: As noted in our response to reviewer 1, we have revised the limitations to specifically note the limitation of omitting frontline providers and patients from the scope of this project. We have also revised the limitations to acknowledge the possibility of sampling bias given the case of participant nonresponse.**

5. Minor Editorial Suggestions
In Table 2, the quote from Participant 8 appears cut off mid-sentence (“President)”). Please verify transcription accuracy.

**Author Response: The authors understand the reviewer’s concern that the quotation appears incomplete. It was transcribed accurately and verbatim but may be confusing as worded.**

**Action Taken: More of the participation quotation was included, with explanatory collateral in brackets, to clarify the meaning and importance of the quotation.**

Ensure all acronyms (e.g., ACO, ERIC) are defined at first use.

**Author Response: The authors thank the reviewer for catching this omission**

**Action Taken: The manuscript has been revised so that all acronyms are spelled out at their first use.**

The phrase “Implementation Science: Health System Science” in Keywords uses a colon; consider using a semicolon or listing as separate terms per journal style.

**Author Response: The authors regret this typographical error and thank the reviewer for catching it.**

**Action Taken: The referenced keywords have been separated with a semicolon.**
